# Disproportionately higher asthma risk and incidence with high fructose corn syrup, but not sucrose intake, among Black young adults: the CARDIA Study

**DOI:** 10.1017/S1368980025000370

**Published:** 2025-03-26

**Authors:** Luanne Robalo DeChristopher, Katherine L Tucker

**Affiliations:** 1 Independent Researcher, Hopewell Life Sciences, M.Sc. Biochemistry, Molecular Biology; 2 Department of Biomedical and Nutritional Sciences, University of Massachusetts Lowell, Lowell, MA, USA

**Keywords:** African Americans, Asthma, High fructose corn syrup, Dysbiosis, Glycation, Fructositis, Fructose malabsorption

## Abstract

**Objective:**

There have been *unsafe levels of unpaired fructose* in the high fructose corn syrup (HFCS) in US beverages, and research/case study evidence shows that their intake is associated with greater asthma prevalence/risk/incidence, a debilitating disease, likely due to fructose malabsorption, gut fructosylation and gut dysbiosis mechanisms. The ‘unexplained’ asthma epidemic has disproportionately affected children and Black individuals, groups with higher fructose malabsorption prevalence than others, and research to assess disproportionately higher asthma risk/incidence among Black individuals in association with HFCS-sweetened beverage intake is lacking.

**Design:**

Demographic, lifestyle and dietary data collected at enrollment (1985–86), and incident asthma through exam 5 (1995–96), were used in Cox proportional hazards models to assess HFCS intake associations (hazard ratios) with asthma risk/incidence.

**Setting:**

CARDIA study participants from Birmingham, AL, Chicago, IL, Minneapolis, MN and Oakland, CA.

**Participants:**

1998 Black and 2104 White young adults.

**Results:**

HFCS-sweetened beverage intake > once/week *was* significantly associated with higher asthma risk relative to ≤ once/week (*P*-trend = 0·04), among Black participants only; risk was 2·8 times higher among 2–4 times/week consumers (HR = 2·8, 95 % CI 1·1, 7·3, *P* = 0·04) and 3·5 times higher when consumed multiple times/d, independent of *sucrose* intake/obesity/dietary quality/smoking/in-home smoke-exposure (HR = 3·5, 95 % CI 1·3, 9·9, *P* = 0·02). Intake of *orange juice*, with nominal unpaired fructose, was *not* associated with asthma in either group, *nor was intake of sucrose*, a disaccharide (paired) of fructose/glucose.

**Conclusions:**

Ubiquitous HFCS in the US food supply, with HFCS that contains high/unsafe unpaired fructose, also known as excess-free-fructose, and the fructose/gut/lung/axis are overlooked risk factors in the ‘unexplained’ US asthma epidemic that disproportionately affects Black individuals.

High fructose corn syrup (HFCS)-sweetened beverage intake is associated with increased asthma prevalence/incidence/risk^(1–14)^ and with many of its comorbidities^(15–17)^. There is consistent evidence that the link is due to its unpaired fructose, which occurs when the fructose-to-glucose ratio exceeds 1:1^(1–7)^. In HFCS-sweetened beverages, this ratio has been higher (1·9:1^(18)^ and 1·5:1^(19)^) than generally recognised as safe (1·2:1)^(20)^. Case study^(21)^ motivated research, that distinguished beverages by their unpaired fructose content, showed that, in addition to HFCS-sweetened beverages, even moderate intake of apple juice, a 100 % juice with a higher (2·2:1) fructose-to-glucose ratio than HFCS, i.e. high unpaired fructose (8 g/250 ml)^(22)^, was associated with increased asthma prevalence/risk/incidence^(1–5)^, whereas 100 % orange juice (orange juice) – a juice with comparable total sugars, total fructose and glycaemic load as apple juice, but nominal unpaired fructose (0·4 g/250 ml)^(22)^, appeared protective.

Unpaired fructose triggers fructose malabsorption, whereas paired fructose/glucose is readily absorbed^(23–26)^. Mounting evidence indicates that unpaired fructose triggers gut formation of asthma-triggering immunogens by inducing gut fructosylation/modification of partially digested dietary proteins and gut hormones/incretins (GIP, GLP-1)^(21,27–30)^, and by inducing changes in the gut microbiome (dysbiosis) which produces asthma-triggering metabolites^(25,31–33)^. Average per capita unpaired fructose intake from HFCS began exceeding dosages (5–10 g) that trigger fructose malabsorption in the early 1980’s, the start of the epidemic. There are striking parallels between the proliferation of HFCS in the US food supply and the ‘unexplained’ US asthma epidemic^(1)^.

Asthma prevalence *more than doubled across age groups* between 1980 and 2004^(34)^ and continued to climb. The increase was greater among Black (3·4–9·1 %) than White individuals (3·1–7·0 %). By 2019, asthma prevalence was 11·2 % among Black *v*. 7·6 % among White individuals^(35)^. Limited research shows that, at comparable intakes, Black individuals have *higher* fructose malabsorption prevalence than others^(36)^. US sugar-sweetened beverages (SSB) are nearly exclusively sweetened with HFCS^(37)^, and Black individuals consume more ‘SSB’ than White individuals^(38)^. Studies have not analysed HFCS and asthma associations by race to assess potential racial disparities attributable to HFCS.

The objective of this study was to test the hypothesis that consumption of HFCS-sweetened beverages is associated with *higher* asthma risk/incidence among Black young adults, and at *lower* intake levels, relative to White young adults, independent of known risk factors. We hypothesised that regular intake of orange juice – a 100 % juice with similar *total sugars (21 g/250 ml), and total fructose (11 g/250 ml)* ^(22)^, as cola (*total sugars 26 g/250 ml* ^(22)^, *total fructose 16–17 g/250 ml*)^(1,18,19)^, but a 1:1 fructose-to-glucose ratio, i.e. nominal unpaired fructose (0·4 g/250 ml)^(22)^ may be protective against asthma across races.

## Methods

### Study design

The Coronary Artery Risk Development in Young Adults (CARDIA) Study examines the development and determinants of clinical and subclinical cardiovascular diseases and their risk factors^(39)^. It began in 1985–1986, with a group (*n* 5115) of Black and White men and women aged 18–30 years (mean age 24·5 years). Participants were selected so that there would be approximately the same numbers in subgroups of race, gender, education (high school or less and more than high school) and age (18–24 and 25–30) in each of four centers: Birmingham, AL; Chicago, IL; Minneapolis, MN and Oakland, CA.

The CARDIA study is uniquely suited to test our hypothesis, as enrollment began (1985–1986), shortly after the time (1980–1984) when US soft drink manufacturers switched from the use of sucrose to HFCS^(37)^. By the start of the study, major US beverage producers, PepsiCo and the Coca-Cola Company, had announced (1984) ‘exclusive’ and ‘up to 100 % use’ of HFCS in all their bottled, canned and fountain drinks^(37)^. Beverages are the major contributors of HFCS to the American diet – by a wide margin^(38)^. For this analysis, we used CARDIA demographic, lifestyle and dietary intake data collected at enrollment (1985–1986) and incident asthma through exam 5 (1995–1996). Consumption frequency of ‘sugary drinks’ is highest between the ages of 20–39 years^(38)^. The age span of CARDIA participants, from enrollment (mean age 24·5 years) through exam 5 (35 years), corresponds with peak adult consumption. We also examined the intake of 100 % citrus juices (orange juice).

We did not include 100 % non-citrus juice intake in our analysis, as intake of apple juice – a 100 % juice with high unpaired, also known as excess-free-fructose (EFF) (7·7 g /250 ml), was not distinguished from other 100 % non-citrus juices/blends, i.e. grape (EFF 1·4 g/250 ml), pineapple (2·1 g EFF/250 ml), etc., that naturally contain nominal/low unpaired/excess-free-fructose^(22)^. The co-mingling of high EFF apple juice, with low EFF non-citrus juices, could render results with ‘100 % non-citrus juices’ difficult to interpret.

**Figure 1.**
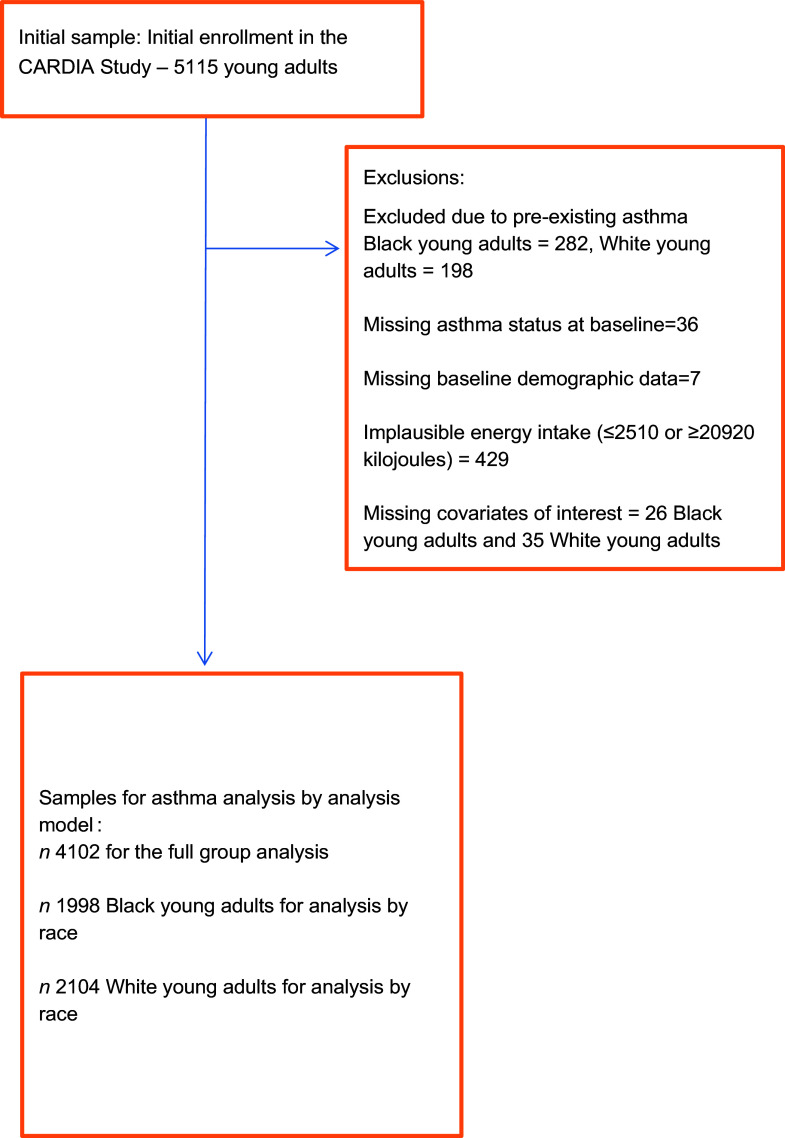
Flow chart showing exclusions and sample sizes.

We conducted survival analysis by race, using Cox regression models for participants with *no* history of asthma at enrollment. Of the 5115 CARDIA participants who enrolled, 282 Black and 198 White participants were excluded due to pre-existing asthma, thirty-six additional were excluded due to missing asthma status at baseline, seven due to missing baseline demographic data and 429 due to implausible energy intake, defined as mean total daily energy intake ≤ 2510 or ≥ 20 920 kJ, leaving 4163 participants for analysis (2024 Black and 2139 White). Of the 2024 Black adults, twenty-six were excluded, and of the 2139 White adults, thirty-five were excluded due to missing covariates of interest. After exclusions, there were 4102 young adults (1998 Black/2104 White) with available data for the study questions of interest (Figure 1).

### Beverage intake

Exposure variables included self-reported intake of HFCS-sweetened beverages, defined as any combination of non-diet soft drinks, and non-diet fruit drinks which, in addition to HFCS, are also sweetened with apple juice. We also analysed 100 % citrus/orange juice intake. Dietary intake data were obtained from a dietary history that included a short questionnaire regarding general dietary practices followed by a FFQ about the typical intake of foods using the previous month as a reference for recall. Both were administered at enrollment^(40)^. CARDIA participants were asked, ‘Do you usually drink any fruit or vegetable juices? How much do you usually have? How often? Responses were distinguished by type (sweetened/unsweetened/low calorie). Do you drink Hi C, Kool-Aid, lemonade or similar types of beverages? How much do you usually have? How often? Do you usually drink Coke, soda or pop? How much do you usually have? How often?’ Responses were distinguished by type (sweetened/unsweetened/low calorie). Volume was provided as cups or ounces and intake frequency as daily, monthly or weekly. The data, as provided by CARDIA, were standardised to cups/d. Intake of any combination of HFCS-sweetened beverages (non-diet soda and non-diet fruit drinks) was divided into ordered quintiles; and intake of 100 % citrus juice, a less frequently consumed beverage, was divided into ordered quartiles. Baseline nutrient analyses of dietary data from the CARDIA study showed that dietary history/intake data provided estimates that agreed reasonably well with expected energy intake for BMI, according to the age and sex-specific Recommended Dietary Allowances^(40)^. This is consistent with research which found a good correlation between the reported frequency of food and food group consumption and the probability of consumption on 24-hour dietary recalls. A small validation study (64 participants) showed that the reliability and comparative validity of the dietary history survey method was higher among White than Black participants^(41)^.

### Ascertainment of endpoints

The outcome variable, asthma status, was self-reported and asked, ‘have you ever had asthma?’ When exam data included asthma status, but the exam date was missing, the next available follow-up date was used, as follow-up contact was made approximately every six months between exams^(39)^.

### Statistical analysis and potential confounders

**Figure 2.**
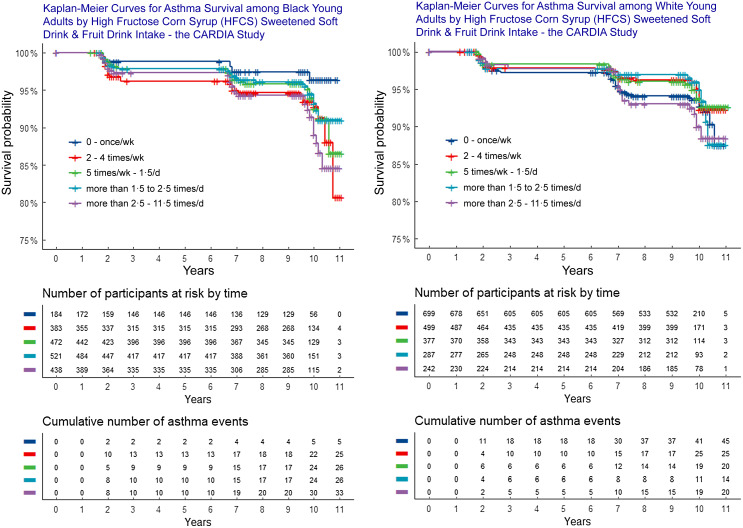
Kaplan–Meier curves of asthma by HFCS sweetened beverage intake among Black and White young adults.

Three Cox proportional hazards models, with time in the study as the time scale, were used for analysis. Proportional hazards assumptions were assessed using Schoenfeld and scaled Schoenfeld residuals for the models (*P* ≥ 0·05) and Kaplan–Meier survival curves for each predictor. Survival curves by race are included in Figure 2. We examined incident asthma over approximately 10 years of follow-up using multivariable-adjusted Cox proportional hazards models to estimate hazard ratios. Person-time was calculated from enrollment (1985–1986) through follow-up to incident asthma, loss to follow-up, death or end of exam 5 (1995–1996), whichever came first. R and Rstudio version 1.3.1093 were used, and a two-tailed *P* ≤ 0·05 with 95 % CI that did not include 1 was considered statistically significant.

Potential confounders were selected based on existing research^(2–4)^. Model 1 included the following potential confounders obtained at enrollment: age, sex, total energy intake (in quartiles), education level – a measure of socioeconomic status (≤ high school graduate or equivalency, *v*. ≥ some college/college graduate), smoking (past, never, current), hours exposed to in-home smoke (continuous), BMI (≤ recommended, overweight, obese), weekly physical activity history score, as determined by a questionnaire that included physical activity type, frequency, duration and intensity of physical activities a participant engaged in, measured in arbitrary ‘exercise units’, as described in detail elsewhere (in quartiles)^(42)^, fruit and vegetable intake, as provided, were normalised to servings/d (continuous), asked as ‘Do you eat fresh fruit? Looking at this list, which ones do you eat and how often? What is your usual serving size for fresh fruit?’; the same questions were asked about canned, cooked, frozen and dried fruit. Vegetable intake was obtained by asking, ‘Do you eat (fresh, frozen and/or canned) vegetables? How much do you usually have? Looking at this list, which of these vegetables do you eat and how often?’; fast food intake/visit frequency (continuous), was asked as ‘How often do you eat breakfast, lunch or dinner out in a place such as McDonalds, Burger King, Wendys, Arbys, Pizza Hut, or Kentucky Fried Chicken.’ Responses were standardised to fast-food visits/week.

Model 2 included further adjustments for other beverage intakes. For example, analysis of any combination of HFCS-sweetened beverages included orange juice as a potential confounder. Model 3 included further adjustments for sucrose intake. Daily sucrose intake was derived from responses to the diet history questionnaire/FFQ and was standardised to tsp/d.

Two Chi-square tests for homogeneity were conducted: (1) with participants lost-to-follow-up (Table 1) and (2) exclusions due to pre-existing asthma (Table 2). Exclusion and loss-to-follow-up homogeneity were assessed by analysing HFCS-sweetened beverage intake by intake quintile and race, to assess potential exclusion and loss-to-follow-up biases that may have affected our results. Loss-to-follow-up was defined as participants who did not participate in the last two of five exams, i.e. exams 4 and 5.

**Table 1.** Chi-square comparison of differences in the distribution of participants lost-to-follow-up, by high fructose corn syrup (HFCS)-sweetened beverage intake and race – the CARDIA^*^ study

Intake frequency of HFCS^†^ sweetened beverages	Black young adults lost-to-follow-up *n* 314 (15·7 % of 1998)	Black young adults proportions lost-to-follow-up (%)	White young adults lost-to-follow-up *n* 199 (9·5 % of 2104)	White young adults proportions lost-to-follow-up (%)
≤ once/week	31 (63·0)	9·9	72^‡^ (40·0)	36·2
2–4 times/week	52 (63·6)	16·6	52 (40·3)	26·1
5 times/week–1·5/d	63 (55·1)	20·1	27 (34·9)	13·6
> 1·5–2·5 times/d	84 (68·6)	26·7	28 (43·4)	14·1
> 2·5–11·5 times/d/(Black young adults) > 2·5–7·5 times/d/(White young adults)	84 (63·7)	26·7	20 (40·3)	10·0

* Coronary artery risk development in young adults study.

† High fructose corn syrup.

‡ Observed and (expected) number. Pearson’s Chi-squared test *P* < 0·00001***, highly significant.

**Table 2.** Chi-square comparison of differences in the distribution of participants excluded due to pre-existing asthma^*^, by high fructose corn syrup (HFCS)-sweetened beverage intake and race – the CARDIA^†^ study

Intake frequency of HFCS^‡^-sweetened beverages	Black young adults *n* 282 (10·8 % of 2620)	Black young adults proportions with pre-existing asthma (%)	White young adults *n* 198 (8·1 % of 2456)	White young adults proportions with pre-existing asthma (%)
≤ once/week	32 (56·4)^§^	11·3	64 (39·6)^§^	32·3
2–4 times/week	47 (56·4)	16·7	49 (39·6)	24·8
5 times/week–1·5/d	64 (56·4)	22·7	32 (39·6)	16·2
> 1·5–2·5 times/d	67 (56·4)	23·8	29 (39·6)	14·6
> 2·5–11·5 times/d (Black young adults)/> 2·5–7·5 times/d (White young adults)	72 (56·4)	25·5	24 (39·6)	12·1

* Asthma status was self-reported and asked as, ‘have you ever had asthma?’ When exam data included asthma status but the exam date was missing, the next available follow-up date was used, as follow-up contact was made approximately every 6 months between exams^(39)^.

† Coronary artery risk development in young adults study.

‡ High fructose corn syrup.

§ Observed and (expected) number. Pearson’s Chi-squared test *P* < 0.00001***, highly significant.

## Results

### Asthma incidence

There were 115 new asthma cases among Black and 124 new asthma cases among White participants over the 10-year follow-up.

### Exclusions for pre-existing asthma

The Chi-square test of homogeneity, by HFCS-sweetened beverage intake frequency and race, among those *excluded from survival analysis* due to pre-existing asthma was significant (*P* < 0·001). Pre-existing asthma prevalence was disproportionately higher among Black (*n* 282), particularly among Black daily HFCS-sweetened beverage consumers than among White participants (*n* 198), which contributed to lower asthma incidence among Black young adults over the 10-year follow-up. Of the 2280 Black and 2222 White participants with plausible total energy intake and non-missing variables of interest at enrollment, 12·4 % and 8·9 %, respectively, were excluded due to pre-existing asthma. Of the Black young adults excluded from analysis due to *pre-existing* asthma (*n* 282), there was a stepwise *increase* with *increasing* HFCS-sweetened beverage consumption from 11·3 % among seldom/never (≤ once/week) to 25·5 % among daily consumers. Results were inverted among White young adults (*n* 198). There was a stepwise *decrease* with *increasing* intake of HFCS-sweetened beverages, from 32·3 % among seldom/never (≤ once/week) to 12·1 % among daily (more than 2·5–7·5 times/d) White consumers (Table 2). Disproportionate exclusions due to pre-existing asthma, by race and HFCS-sweetened beverage intake, contributed to exclusion bias and likely lowered asthma incidence/risk among Black participants.

### Loss-to-follow-up

The Chi-square test of homogeneity, by HFCS-sweetened beverages intake frequency and race, of participants *lost-to-follow-up*, was significant (*P* < 0·001). Loss-to-follow-up was disproportionately higher among Black, i.e. 15·7 % (*n* 314), than White, i.e. 9·5 % (*n* 199) participants and more than 56·9 % of Black participants lost-to-follow-up were *daily* consumers of HFCS-sweetened beverages, *v*. 27·1 % of White participants (Table 1). Disproportionate loss-to-follow-up among Black daily HFCS-sweetened beverage consumers may have contributed to loss-to-follow-up bias and *understatement* of asthma risk and incidence among Black participants.

### Participant characteristics at enrollment

Mean age at enrollment was 24·5 years. Obesity (18·0 *v*. 6·6 %) and current smoking (31·7 *v*. 25·5 %) were higher among Black than White participants, respectively. The percentage of participants with at least some college was lower among Black (51·2 %) than White young adults (74·4 %). (Table 3). Post hoc analysis showed that higher education was associated with less frequent/never HFCS-sweetened beverage intake, i.e. two times per week or less, among White, but not among Black participants (data not shown). A higher percentage of Black participants consumed HFCS-sweetened beverages regularly (5 or more times/week) (71·6 %) than White participants (43 %), respectively, whereas orange juice intake was similar across races. Approximately half of the participants consumed orange juice six or more times/week.

**Table 3.** Baseline characteristics of young adults by race – the CARDIA study

	Black young adults (*n* 1998)			White young adults (*n* 2104)		
	%	Mean	sd	%	Mean	sd
Age (years)		24·5	3·8		25·5	3·3
Gender (Female)	61·0			55·3		
BMI^2^		25·4	5·6		23·6	3·8
Overweight (25–29·9 kg/m)^2^	25·7			22·3		
Obese (30+ kg/m^2^)	18·0			6·6		
Total energy intake (kJ/d)		11 021	4322		10 360	3925
History of smoking						
Ex-smoker	9·2			18·8		
Current smoker	31·7			25·5		
Hours exposed to in-home smoke		12·5	20·0		9·1	17·8
Education level						
≤ 12 years (high school grad/graduate equivalency)	48·7			25·6		
13+ years (≥ some college/college graduates)	51·2			74·4		
Physical activity score		23·5	20·3		21·7	18·1
Self-reported hypertension (yes)	10·7			7·7		
Self-reported high cholesterol/dyslipidaemia (yes)	1·7			2·4		
Self-reported diabetes (yes), *n* 1978	1·2			0·7		
Serum TAG, *n* 1977						
Normal (normal (< 150 mg/dl)	96·8			92·8		
Borderline high (150–199 mg/dl)	2·0			4·1		
High (≥ 200 mg/dl)	1·2			3·0		
Teaspoons of sucrose /d		13·7	11·1		10·5	9·9
Fast food frequency of visits/week		2·0	2·0		1·8	2·3
Fruit intake frequency servings/d		3·2	2·9		2·8	2·2
Vegetable intake frequency servings/d		2·0	1·7		3·0	2·2
Intake frequency of any combination of HFCS sweetened beverages (non-diet soda, non-diet fruit drinks)						
≤ once/week	9·2			33·2		
2–4 times/week	19·2			23·7		
5 times/week–1·5 times/d	23·6			17·9		
> 1·5–2·5 times/d	26·1			13·6		
> 2·5–11·5 times/d (Black) / > 2·5 – 7·5 times/d (white)	21·9			11·5		
Intake frequency HFCS sweetened non-diet soda						
≤once/month	14·7			37·2		
2 times/month–3 times/week	27·7			25·2		
4 times/week–1·5 times/d	31·2			19·5		
> 2–7·5 times/d (Black) / 2 - 6 times/d	26·3			18·1		
Intake frequency HFCS sweetened non-diet fruit drinks						
Zero/ none	23·7			45·1		
A few times/year–2 times/week	31·3			37·7		
3 times/week–8/d (Black) / 3 times/week - 3 times/d (White)	45·0			17·2		
Intake frequency 100 % citrus juices (orange juice)						
≤ 2 times/week	24·9			27·1		
3–5 times/week	24·8			27·1		
6 times/week–1·5 times/d	24·6			25·5		
2–8 times/d (Black) / 2–6 times/d (White)	25·7			20·3		
Intake frequency 100 % non-citrus juices						
Zero/none	22·3			29·2		
A few times/year–2 times/week	22·9			29·2		
3–6 times/week	25·9			24·4		
1–8 times/d (Black) / 1–4 times/d (White)	28·9			17·2		

### Relationship with asthma

Asthma risk was associated with HFCS-sweetened beverage intake among Black young adults only. Associations were not significant (NS) among White participants (Tables 4 and 5 and Figure 2). Black men and women who consumed any combination of HFCS-sweetened beverages (non-diet soda and fruit drinks) two or more times per week had significantly higher asthma risk relative to seldom/never consumers (once/week or less) (*P* for trend = 0·04), independent of sex, BMI, age, physical activity, hours exposed to in-home smoke, smoking, education, total energy intake, fast-food visits/week and fruit and vegetable intake. Increased risk *remained significant* after further adjustments for 100 % orange juice and *sucrose* intake (Table 4).

**Table 4.** Asthma^†^ relative risks and incidence by beverage consumption among Black young adults, the CARDIA^‡^ study

		Model 1				Model 2				Model 3							
Cox proportional hazards Hazard ratios (HR) *n* 1998	No.	HR	95 % CI	*P*	*P* of trend	HR	95 % CI	*P*	*P* of trend	HR	95 % CI	*P*	*P* of trend	*#* of cases	IR per 1000	Person time years	Cases/1000/year
**Any combination of HFCS-^||^ sweetened drinks/any combination of non-diet soda and non-diet fruit drinks**		HR^§^– adjusted for demographic, lifestyle, dietary factors and BMI				HR – further adjusted for intake of 100 % citrus juices (orange juice)^¶^				HR – further adjusted for sucrose** intake (tsp/d)							
≤ once/week	184	Reference				Reference				Reference				5	27·2	1456	3·4
2–4 times/week	**383**	**2·89**	**1·09, 7·61**	**0·03** ^*^		**2·74**	**1·04, 7·21**	**0·04** ^*^		**2·76**	**1·04, 7·27**	0·04*		**25**	**65·3**	**3081**	**8·1**
5 times/week–1·5/d	472	2·19	0·83, 5·77	0·11		2·06	0·78, 5·43	0·14		2·09	0·79, 5·50	0·14		26	55·1	3873	6·7
> 1·5–2·5 times/d	521	2·20	0·82, 5·88	0·11		2·18	0·81, 5·80	0·12		2·24	0·83, 6·03	0·11		26	49·9	4111	6·3
> 2·5–11·5 times/d	438	**3·35**	**1·26, 8·92**	**0·02** ^*^	**0·04** ^*^	**3·30**	**1·24, 9·31**	**0·02** ^*^	**0·04** ^*^	**3·55**	**1·26, 9·94**	**0·02** ^*^	**0·04** ^*^	**33**	**75·3**	**3292**	**10·0**
**100 % citrus juice (orange juice)^¶^**		HR^§^ – adjusted for demographic, lifestyle, dietary factors and BMI				HR – further adjusted for HFCS|| sweetened beverage intake				HR – further adjusted for sucrose** intake (tsp/d)							
≤ 2 times/week	498	Reference				Reference				Reference				29	68·0	3920	7·4
3–5 times/week	496	1·00	0·60, 1·69	0·99		0·99	0·59, 1·67	0·97		0·99	0·59, 1·66	0·96		29	50·5	3975	7·3
6 times/week–1·5/d	491	1·08	0·64, 1·83	0·77		1·10	0·65, 1·87	0·71		1·10	0·65, 1·86	0·72		34	54·5	3946	8·6
> 1·5–8 times/d	513	0·61	0·33, 1·15	0·12	0·19	0·62	0·33, 1·18	0·14	0·20	0·62	0·33, 1·17	0·14	0·20	23	62·7	3972	5·8

Hazard ratios, their 95 % CI and *P* values are shown. Boldface signifies statistical significance at a glance.

* Indicates statistical significance, i.e. *P* ≤ 0·05.

† Data exclude pre-existing asthma at enrollment (1985–1986). Asthma incidence reflects new cases from enrollment through approximately 10 years of follow-up.

‡ Coronary Artery Risk Development in Young Adults Study.

§ Hazard ratio is adjusted for sex, BMI, age, physical activity, smoking, education, hours exposed to in-home smoke, fruit, vegetable and total energy intake and fast food frequency of visits/week.

|| The unpaired fructose in HFCS has been higher than generally recognised as safe, 5–9 g/250 ml, as measured by independent labs^(1,18,19)^.

¶ Orange juice is the most consumed 100 % citrus juice – a juice with low excess-free-fructose (0·4 g/250 ml).

** Sucrose – also known as table sugar is a disaccharide of fructose and glucose, i.e. has a 1:1 fructose-to-glucose ratio and contains no unpaired fructose.

**Table 5.** Asthma^†^ relative risks and incidence by beverage consumption among White young adults, the CARDIA^‡^ study

		Model 1				Model 2				Model 3							
Cox Proportional Hazards Hazard ratios (HR) *n* 2104	No.	HR	95 % CI	*P*	*P* of trend	HR	95 % CI	*P*	*P* of trend	HR	95 % CI	*P*	*P* of trend	*#* of cases	IR per 1000	Person time years	Cases/1000/year
**High fructose corn syrup (HFCS)^||^ sweetened drinks/any combination of non-diet soda and non-diet fruit drinks**		HR^§^ – adjusted for demographic, lifestyle, dietary factors and BMI				HR – further adjusted for intake of 100 % citrus juices (orange juice)^¶^				HR – further adjusted for sucrose** intake (tsp/d)							
≤ once/week	699	Reference -------				Reference -------				Reference -------				45	64·4	5943	7·6
2–4 times/week	499	0·81	0·49, 1·33	0·41		0·79	0·48, 1·31	0·37		0·78	0·47, 1·28	0·33		25	50·1	4325	5·8
5 times/week–1·5/d	377	0·90	0·52, 1·57	0·71		0·89	0·51, 1·55	0·68		0·82	0·47, 1·46	0·50		20	53·1	3369	5·9
> 1·5–2·5 times/d	287	0·85	0·45, 1·62	0·62		0·88	0·46, 1·68	0·70		0·77	0·38, 1·53	0·45		14	48·8	2419	5·8
> 2·5–7·5 times/d	242	1·21	0·64, 2·30	0·55	0·57	1·25	0·66, 2·39	0·49	0·47	0·86	0·34, 2·14	0·74	0·77	20	80·1	2080	9·6
**100 % Citrus juice adjusted (orange juice)^¶^**		HR^§^ – adjusted for demographic, lifestyle, dietary factors and BMI				HR^§^ – adjusted for demographic, lifestyle, dietary factors and BMI				HR – further adjusted for HFCS^||^ sweetened beverage intake							
≤ 2 times/week	571	Reference -------				Reference -------				Reference -------				36	63·0	4919	7·3
3–5 times/week	571	1·26	0·79, 1·99	0·33		1·29	0·81, 2·04	0·28		1·32	0·83, 2·10	0·24		40	70·1	4923	8·1
6 times/week–1·5/d	535	1·12	0·67, 1·86	0·67		1·14	0·68, 1·90	0·62		1·16	0·70, 1·94	0·56		33	61·7	4629	7·1
2–6 times/d	427	0·74	0·36, 1·51	0·41	0·38	0·74	0·36, 1·50	0·41	0·37	0·77	0·38, 1·56	0·46	0·42	15	35·1	3664	4·1

Hazard ratios, their 95 % CI and *P* values are shown.

*Indicates statistical significance, i.e. *P* ≤ 0·05. ∼ signifies results that approached statistical significance.

† Data exclude pre-existing asthma at enrolment (1985–1986). Asthma incidence reflects new cases from enrolment through approximately 10 years of follow-up.

‡ Coronary Artery Risk Development in Young Adults Study.

§ Hazard ratio is adjusted for sex, BMI, age, physical activity, smoking, education, hours exposed to in-home smoke, fruit, vegetable, total energy intake and fast food frequency of visits/week.

|| The unpaired fructose in HFCS has been higher than the generally recognised as safe, 5–9 g/250 ml, as measured by independent labs^(1,18,19)^.

¶ Orange juice is the most consumed 100 % citrus juice – a juice with low excess-free fructose (0·4 g/250 ml).

** Sucrose – also known as table sugar is a disaccharide of fructose and glucose, i.e. has a 1:1 fructose-to-glucose ratio and contains no unpaired fructose.

Among Black participants, asthma risk ranged from 2·75 times higher among 2–4 times/week HFCS-sweetened beverage consumers (hazard ratio 2·76, 95 % CI 1·04, 7·27, *P =* 0·04) to 3·5 times higher among > 2·5 times/d consumers (hazard ratio 3·55, 95 % CI 1·3, 9·9, *P =* 0·02), relative to ≤ once/week (Table 4). Among Black participants, asthma incidence was 177 % higher, among *daily* HFCS-sweetened beverage consumers (75·3/1000) *v*. ≤ once/week (27·2/1000). There was a 195 % increase in the number of new asthma cases/1000/year among Black participants, increasing from 3·4/1000/year among ≤ once/week consumers to 10/1000/year among 2·5 or more times/d consumers – a nearly three-fold increase (Table 4 and Figure 2). Given the disproportionately higher number of Black participants excluded from the analysis due to loss-to-follow-up, particularly daily HFCS-sweetened beverage consumers, asthma risk and incidence associated with HFCS-sweetened beverage intake among Black young adults may be understated due to loss-to-follow-up bias (Table 1). Moreover, more Black participants were excluded from the analysis due to pre-existing asthma, which contributed to fewer incident asthma cases among Black, relative to White, participants. Notably, exclusions *increased* stepwise with *increasing* HFCS-sweetened beverage consumption, among Black participants only (Table 2).

## Discussion

The ‘unexplained’ US asthma epidemic (∼1980–present) occurred *after* air quality improvements, due to the passage and expansion of the Clean Air Act (1970). It occurred *after* stronger occupational safety and worker protections, due to the passage and expansion of the Occupational Safety and Health Act (1970). It has *parallelled a decline* in smoking rates across races^(43)^, and according to the US Centers for Disease Control (CDC) has occurred among *normal weight*, not overweight/obese individuals^(44)^. The start of the epidemic is *not* plausibly attributable to family history, as family history *did not change* coincident with the start of the epidemic. The most affected by the epidemic have been children and Black people^(34,35,43)^ – groups with higher fructose malabsorption rates at lower unpaired fructose/excess-free-fructose intake than others^(1,36)^. Black young adults who consumed HFCS-sweetened beverages regularly had significantly higher asthma risk/incidence even at moderate intake (twice per week), relative to less frequent/never consumers. Asthma risk was 2·8 times higher when consumed 2–4 times per week, and 3·5 times higher when consumed multiple times/d, independent of sex, BMI, age, physical activity, education, hours exposed to in-home smoke, smoking, total energy, fruit, vegetable, fast food and *sucrose* intake. *Results showed a dose response relationship between increasing HFCS sweetened beverage intake and asthma risk/incidence, among Black young adults only.* There was *no asthma association with orange juice or sucrose*, at any intake level, across any of the analysis models, which supports our hypothesis that the association is with the high/unsafe fructose-to-glucose ratios in HFCS-sweetened beverages, not with sucrose – a disaccharide of fructose and glucose, nor with paired fructose as occurs in orange juice. Results support our hypothesis that Black individuals have *higher* asthma risk/incidence at *lower* HFCS-sweetened beverage intake than White individuals. The 177 % and 195 % increases in asthma incidence and cases/1000/year, among Black young adults, between less frequent/never consumers of HFCS-sweetened beverages and weekly/daily consumers are remarkable. It is also remarkable that exclusions for pre-existing asthma *increased* stepwise with *increasing* HFCS-sweetened beverage consumption, among Black participants only.

Results are consistent with the hypothesis that individuals with fructose malabsorption are at increased risk of unabsorbed, unpaired fructose-induced asthma. These results add to the growing body of evidence that links HFCS-sweetened beverage intake with increased asthma prevalence/risk/incidence^(1–13)^ and with protective effects from orange juice^(2–4,6,8)^. In a study with mostly White adults^(4)^, 2–4 times/week intake of HFCS sweetened beverages was associated with 1·5 times higher asthma risk/incidence than less frequent/never, i.e. lower than the 2·75 times higher asthma risk seen among Black young adults herein at comparable intakes. These CARDIA results resemble prospective research with children, wherein those who consumed HFCS-sweetened beverages weekly and more than once/d had two times, and nearly three times higher asthma risk, than 2·5 times/week or less consumers^(3)^. Asthma risks were also higher with 100 % juice intake, which, for children, is mainly apple juice. Results are consistent with the fact that children are at higher risk of fructose malabsorption’s health consequences at lower intakes than adults^(1–3,6–10,23,24)^. These studies build upon research with nationally representative data, wherein children ages 2–9 years had higher asthma prevalence with increasing intake of HFCS-sweetened beverages and apple juice, and orange juice intake appeared protective^(2)^. Similar research with high schoolers and adults, by the US CDC, found that HFCS-sweetened beverage intake was associated with higher asthma prevalence^(10,11)^. Researchers hypothesised that the association may be with the preservatives in soft drinks, but subsequent research concluded that there is *no scientific* evidence that preservatives in US soft drinks are associated with asthma. Diet soft drinks contain the same preservatives and, in the United States, diet soft drink intake is *not* associated with asthma^(5)^.

HFCS is not exclusive to beverages. One-third of all HFCS consumed in the United States is in food, and there are other manufactured sources of unpaired fructose, including agave syrup (≥ 70–90 % fructose), crystalline fructose and apple juice/powder that contribute to daily exposure to unpaired fructose that have not been accounted for here and elsewhere. Thus, we may be underestimating the role of unpaired fructose exposure in the US asthma epidemic and asthma racial disparities. These results underscore the need for more research on racial differences in fructose malabsorption and its broader health consequences, as research is limited.

From a mechanistic point of view, results are consistent with the unabsorbed unpaired fructose/gut/lung axis in asthma^(21,27–30,32,33)^. Mounting evidence points to unpaired fructose-induced gut dysbiosis in asthma. Gut dysbiosis increases uremic toxins^(45)^ and LPS, which bind asthma mediating receptors (RAGE)^(24,31,32)^, and *lowers* SCFAs^(25)^ which are protective against inflammation. Researchers found that altered gut microbiome compositions were involved in the severity of asthma and that specific bacteria were related to different asthma phenotypes and serum IgE concentration^(46)^. Unabsorbed unpaired fructose, as in HFCS, also *induces* gut formation of immunogens, known as advanced glycation end-products (AGE/FruAGE), by chemically interacting/modifying dietary proteins and gut hormones (GLP-1 and GIP)^(21,27–30)^. Phosphates, from the phosphoric acid in soft drinks, accelerate/catalyse the Maillard reaction (fructosylation, also known as gut glycation by fructose)^(14)^. Results are consistent with research which found that individuals with asthma had *low* concentration of *soluble RAGE* (sRAGE), the receptor isoform that *quenches* RAGE proinflammatory signalling. *Researchers hypothesized that disproportionately higher serum AGE may underlie idiopathic asthma*, particularly in severe and/or persistent asthma^(31,32)^. Gut formation of asthma-triggering immunogens, due to higher fructose malabsorption prevalence among Black people, and high unsafe unpaired fructose in HFCS, plausibly plays a role in asthma racial disparities.

The coherent body of evidence is consistent with research by Brinkley et al, who found that sRAGE was ∼30 % *lower* in Black, compared with White individuals^(47)^. They reasoned that the higher burden of ligand (bad) to soluble receptor (good), i.e. the carboxymethyllysine (CML) to sRAGE ratio among Black participants, supports the possibility that they have either *higher AGE burden* and/or lower sRAGE and, thereby, have less endogenous protection against CML, a type of AGE, which predisposes them to higher risk of cardiovascular, metabolic, neurological and inflammatory diseases. Asthma is an inflammatory disease. They noted that race was the strongest predictor of the CML to sRAGE ratio^(47)^. CML is also found in foods, thus the term dAGE^(14)^. In another study, researchers hypothesised that grilled meat consumers would have elevated serum/urinary CML. Their results, however, pointed to unpaired fructose and the intestines as the source of elevated pro-inflammatory CML/AGE, not the food^(14)^. Their findings are consistent with another study, wherein, vegetarians who consumed *a lot* of apples/apple juice had higher serum AGE than omnivores, providing further evidence that unabsorbed unpaired fructose in the gut forms AGE^(14)^.

Asthma increases the risk of a broad range of respiratory, non-respiratory and inflammatory diseases including inflammatory bowel disease, diabetes, autoimmune diseases and heart disease that are consistent with the systemic disease nature of asthma and its impact beyond the airways^(48)^. These comorbidities are consistent with ramifications of gut fructosylation of dietary peptides and incretins, high immunogen burden (AGE/FruAGE, LPS, uremic toxins), GLP-1 and GIP inactivation and dysregulation and gut dysbiosis. Results herein are consistent with the paediatric case study^(21)^ that motivated this and other research^(1–5,14–17)^, wherein results of a rigorous food elimination diet, the gold standard to assess food sensitivities, showed that HFCS was the unequivocal trigger of severe asthma/dyspnoea/lip cyanosis/chronic bronchitis/abdominal and knee pain^(21)^. Asthma-associated infectious and inflammatory multimorbidities are under-recognised conditions that pose major health threats to people with asthma^(48)^. There are health policy implications, as increased asthma risk among Black participants was evident even at moderate HFCS-sweetened beverage intake, i.e. two times/week. Recommendations to reduce SSB are inadequate, as HFCS, and other high excess-free-fructose sugars (crystalline fructose, agave syrup (70–90 % fructose) and apple powder) are ubiquitous in the US food supply. Studies, including research by the US CDC, showed a link between ‘SSB’ intake and asthma. These studies did not distinguish beverages by sugar type^(7,9–13)^. Research that distinguished beverages by their fructose-to-glucose ratio is consistent^(2–6,8)^. The asthma association is with the high fructose-to-glucose ratio in HFCS, and apple juice, not with paired fructose, and as we see herein, *not* with sucrose.

US CDC messaging to reduce ‘sugary drink’ intake does not address the science that links the unpaired fructose in HFCS with asthma. Messaging has focused on SSB as leading sources of ‘added sugars’ and the need to reduce intake of ‘sugary drinks,’ due to their ‘association with weight gain, obesity, type 2 diabetes, heart disease, kidney diseases, non-alcoholic liver disease, tooth decay and cavities and gout, a type of arthritis.’ There is no mention of the association between HFCS intake and asthma, despite the growing body of research which shows that the association is with the unpaired fructose in HFCS and apple juice. Sucrose intake is not new and does not explain the US asthma epidemic. What *is new* is the unprecedented proliferation of sweeteners with high unsafe unpaired fructose (HFCS, crystalline fructose, agave syrup, apple powder and apple juice) in the US food supply that coincides with the tripling of apple juice intake^(1)^. Between 1980 and 1999, HFCS average per capita intake went from 24 g/d (∼ 1/3 lb/week) to its peak in 1999 of approximately 80 g/d (> 1 lb/week)^(1,49)^, attributable to the expanded use of HFCS across the US food supply and nearly exclusive use of HFCS in beverages^(37)^. The unpaired fructose in HFCS-sweetened cola ranges from 5 to 9 g/250 ml^(1)^, when fructose-to-glucose ratios are high (1·5:1 and 1·9:1), as measured by independent labs^(18,19)^. The unpaired fructose in one can of cola with 65 % fructose/35 % glucose is 12 g, i.e. higher than the dose (5 g/10 g) that triggers fructose malabsorption in children/adults^(23,24)^.

In 1996, the US Food and Drug Administration designated HFCS with 55 % fructose/45 % glucose, a 1·2:1 fructose-to-glucose ratio, as generally recognised as safe^(20)^, which appears too high in the context of fructose malabsorption in young children^(1)^. Industry practice deviates from what is generally recognised as safe. The unpaired fructose dose (5 g) that triggers fructose malabsorption in children was reached in 1980^(1)^. The start of the ‘unexplained’ US asthma epidemic^(34,35,45)^ and the adult dosage (10 g)^(23,24)^ was reached in 1984^(1)^ – the year *before* the start of the CARDIA study, as based on mean per capita HFCS intake at 65 % fructose, i.e. the concentrations measured by independent labs. 1984 is also the year that PepsiCo and the Coca-Cola Company announced ‘exclusive’ use and ‘up to 100 %’ use of HFCS in canned, bottled and fountain drinks^(37)^. By the end of the 10-year follow-up period (1995–1996), average per capita unpaired fructose intake from HFCS was 15 g^(1)^. In 2014, industry-sponsored researchers, using different technology, identified the presence of glucose oligomers in HFCS, not previously identified by independent labs^(50)^. This finding is not relevant in the context of fructose malabsorption, as there is no research, that we know of, wherein higher saccharides aid in unpaired fructose absorption or avert fructose malabsorption and its health consequences.

### Limitations

This study has limitations. First, it may not be generalisable to other population settings, as the CARDIA study is specific to White and Black American young adults living in specific geographic regions, and therefore may not reflect outcomes of White and Black Americans living elsewhere. However, our results are consistent with prospective study data of mostly White adults in the Framingham Heart Offspring Cohort Study^(4)^ and children in the National Children’s Study^(3)^. Results are consistent with many cross-sectional studies of HFCS-sweetened beverage intake and asthma, including with nationally representative data – the US National Health and Examination Survey (NHANES)^(2,5–13)^, and with other large-scale health survey data, including the Youth Risk Behavior Survey^(10)^, the Behavioral Risk Factor Surveillance System^(11)^ and the California Health Interview Survey^(12)^. Second, data were based on a combination of inputs that included self-reports, which are subject to reporting bias. However, associations between the beverages analysed and asthma are consistent with a large body of existing literature^(1–15)^. Third, there were statistically significant racial differences in loss-to-follow-up and exclusions due to pre-existing asthma. Exclusion bias lowered asthma incidence among Black participants. Loss-to-follow-up bias may add to an understatement of asthma racial disparities associated with the consumption of HFCS-sweetened beverages.

### Conclusion

Intake of HFCS-sweetened soda and HFCS/apple juice-sweetened fruit drinks, beverages with high fructose-to-glucose ratios, was associated with disproportionately higher asthma risk and incidence in Black than White young men and women. The ubiquitous presence of HFCS in the U.S. food supply over the past 40 years appears to be contributing to asthma racial disparities, particularly with higher fructose malabsorption prevalence among Black individuals, relative to other groups, unabsorbed unpaired fructose-induced gut dysbiosis, gut formation of advanced glycation end-products and dysregulation and inactivation of GLP-1 and GIP. These mechanisms trigger the formation of asthma-provoking immunogens with far-reaching consequences. Consistent with other studies^(1–15)^, these results provide more evidence of a dose–response relationship between HFCS-sweetened beverage intake and asthma risk/incidence. Results support a role for HFCS-sweetened beverages in asthma racial disparities and the ‘unexplained’ US asthma epidemic that has disproportionately affected Black individuals. More research, more comprehensive nutrition facts, food warning labels, and better food safety oversight are needed.
